# Stromal Cell-Derived Factor-1 Enhances the Therapeutic Effects of Human Endometrial Regenerative Cells in a Mouse Sepsis Model

**DOI:** 10.1155/2020/4820543

**Published:** 2020-03-17

**Authors:** Wang Jin, Yiming Zhao, Yonghao Hu, Dingding Yu, Xiang Li, Yafei Qin, Dejun Kong, Hao Wang

**Affiliations:** ^1^Department of General Surgery, Tianjin Medical University General Hospital, 154 Anshan Road, Heping District, Tianjin 300052, China; ^2^Tianjin General Surgery Institute, Tianjin Medical University General Hospital, Tianjin, China

## Abstract

Endometrial regenerative cells (ERCs) are mesenchymal-like stromal cells obtained from human menstrual blood, whose positive therapeutic effects have been validated in several experimental models. Stromal cell-derived factor-1 (SDF-1), the ligand for CXCR4, plays an important role in the migration of mesenchymal stromal cells. The purpose of this study was to investigate the role of the SDF-1/CXCR4 pathway in the therapeutic effects of ERCs in a mouse sepsis model. Through preexperiment and confirmation, wild-type C57BL/6 mice were intraperitoneally injected with 10 mg/kg lipopolysaccharide (LPS). The therapeutic effects of ERCs with different pretreatments were evaluated by assessing sepsis-related symptoms, detecting tissue damage and measuring levels of inflammatory and oxidative stress-related factors. The *in vitro* experiments demonstrated that there was a much higher CXCR4 expression on ERCs when they were cocultured with SDF-1. The *ex vivo* experiment results showed that SDF-1 expression significantly increased in mouse tissues. Further experiments also confirmed that, compared with the unmodified ERC treatment group, SDF-1 pretreatment significantly enhanced the therapeutic effects of ERCs on alleviating sepsis symptoms, ameliorating pathological changes, reducing Bax level, and increasing Bcl-2 and PCNA expressions in mouse liver tissues. Furthermore, it was also found that SDF-1-pretreated ERCs contributed to reducing the levels of proinflammatory cytokines (TNF-*α*, IL-1*β*) and increasing the levels of anti-inflammatory factors (IL-4, IL10) in mouse serum, liver, and lung. Moreover, SDF-1-pretreated ERCs could also significantly decrease the levels of iNOS and MDA and increase the expression of Nrf2, HO-1, and SOD in liver tissues. Taken together, these results indicate that SDF-1 pretreatment plays a key role in improving the therapeutic effects of ERCs in alleviating sepsis-related symptoms, reducing tissue damage, regulating inflammatory imbalance, and relieving oxidative stress in a mouse sepsis model, which provides more possibilities for the clinical application of ERCs in sepsis and relevant diseases.

## 1. Introduction

Globally, the mortality caused by sepsis continues to be high, but better treatments have not yet been established. The most severe degree of sepsis can lead to acute respiratory distress syndrome (ARDS) and multiple organ dysfunction syndrome (MODS) [1]. The pathophysiology features of sepsis are mainly characterized by inflammatory response imbalance, oxidative stress dysfunction, and excessive apoptosis [2]. Inflammatory response imbalance is mainly caused by excessive production of proinflammatory cytokines such as TNF-*α*, IL-1*β*, and insufficient production of anti-inflammatory cytokines such as IL-4 and IL-10 [2]. In the course of sepsis, lungs and liver are relatively more sensitive to damage factors, and first show damage changes at the molecular and cellular levels [3, 4]. Traditional strategies for sepsis treatment include early identification and diagnosis, effective antibiotic treatment, rapid fluid resuscitation, and supportive treatment such as blood transfusion and ventilation [5]. However, the improvement in efficacy of traditional treatment strategies on sepsis is still limited [5]. Considering the serious destructiveness of sepsis, the necessity of complementing existing treatment strategies is in urgent need.

Mesenchymal stromal cells (MSCs) are one of the most widely studied pluripotent stem cells. Due to their unique immunomodulatory ability and low immunogenicity, MSCs are recognized as an attractive candidate for the treatment of autoimmune diseases and inflammatory diseases [6]. Besides, MSCs were also found with an ability to promote tissue repair by its role in promoting proliferation, stimulating differentiation, reducing apoptosis, and enhancing angiogenesis in local tissues [7]. In 2007, Meng et al. discovered a new type of adult mesenchymal-like stromal cells from menstrual blood, which were called endometrial regenerative cells (ERCs) [8]. They demonstrated that ERCs have the ability to self-regenerate and differentiate into specific cell lines in appropriate medium [8]. It is worth noting that ERCs have many advantages in clinical applications, including noninvasive acquisition methods, sufficient sources, high reproductive rate, multidirectional differentiation potential, immunomodulatory ability, and large-scale amplification without morphological variation [9]. Furthermore, ERCs do not express MHC class I molecules and only express low levels of MHC class II molecules [10], which enables ERCs with low immunogenicity to function smoothly in mice without rejection. Our research group has validated the therapeutic effects of ERCs in a variety of experimental disease models, such as promoting immune tolerance in cardiac allograft [11], relieving ulcerative colitis injury [12], alleviating acute liver injury [13], mitigating pulmonary fibrosis [14], and lightening renal ischemia-reperfusion injury [15]. However, up to now, we and other groups have not found and reported any case of ERCs rejected by mice.

Stromal cell-derived factor-1 (SDF-1) is also known as C-X-C motif chemokine ligand 12 (CXCL12), whose binding receptor is a highly conserved G-protein coupled chemokine receptor C-X-C motif chemokine receptor 4 (CXCR4). It has been reported that SDF-1 is highly conserved during evolution. The similarity between human and mouse in the coding region nucleotide sequence and amino acid sequence of SDF-1 is 97% [16]. In addition, CXCR4 is highly conserved during evolution [17]. The conservation of SDF-1/CXCR4 in different species can ensure that human endometrial regenerative cells can function properly in mice [11]. Furthermore, studies have shown that AMD3100, a potent antagonist of CXCR4 receptor, can effectively block the function of SDF-1/CXCR4 signaling pathway [18].

SDF-1/CXCR4 signaling pathway plays an important role in mediating cells chemotaxis and metastasis [19], inducing angiogenesis [20], and promoting immunoregulation [21]. Studies have shown that ERCs can also secrete high levels of SDF-1, which is 9824 ± 1700 pg/ml in culture supernatant measured by ELISA [11]. Meanwhile, it has been demonstrated that ERCs with high SDF-1 secretion play a positive role in promoting immune tolerance in cardiac allograft [11]. It is worth noting that SDF-1 is generated under certain pathological conditions, including hypoxia, inflammation, ischemia, cancer, and autoimmune diseases [22]. Also, SDF-1/CXCR4 plays an important role in the migration of MSC to the injury sites [23]. More importantly, verified by previous literatures, CXCR4 is considered to express on the surface of ERCs and can be stimulated to a higher expression after ERCs cocultured with SDF-1 [9]. Given previous researches, SDF-1/CXCR4 signaling pathway is expected to play a vital role in affecting ERCs migration, secretion, and immune regulation. We speculate that ERCs with overexpressed CXCR4 could reach the injury sites under chemotaxis by SDF-1 and could secrete SDF-1 to recruit more ERCs to regulate immune imbalance and relieve tissue damage.

Considering previous researches, whether SDF-1-pretreated ERCs could exert a therapeutic effect on sepsis model has not been determined. Hence, the purpose of this study was to verify the therapeutic effects of SDF-1-pretreated ERCs in alleviating sepsis-related symptoms, reducing tissue damage, regulating inflammatory imbalance, and relieving oxidative stress in a mouse sepsis model.

## 2. Materials and Methods

### 2.1. Animals

All animal studies and experimental procedures were conducted on the basis of protocols approved by the Animal Care and Use Committee of Tianjin Medical University (Tianjin, China), according to the Chinese Council on Animal Care guidelines. Male C57B/L6 mice, weighing 22–25 g, 6–8 weeks old, were used in the present study. Animals were bred and housed under a conventional experimental environment with unrestricted access to water and food pellets in the General Surgery Institute.

### 2.2. Preparation of ERCs

Under the ethical approval of Tianjin Medical University (Tianjin, China), human ERCs were isolated from menstrual blood collected from a sterilized menstrual cup on the first day of menstruation in volunteer healthy women (20–30 years old). The culture and amplification of ERCs are identical to the previous description [11]. Briefly, mononuclear cells from menstrual blood were suspended in Dulbecco's modified Eagle's medium supplemented with 10% fetal bovine serum and 1% penicillin/streptomycin. The cell suspension was dispensed into two 10 cm culture dishes and cultured in a 37°C 5% CO_2_ incubator. The cells adhered to the surface of the culture dish after overnight incubation. After 2 weeks of culture, the cells were in a spindle shape, and the estimated number of adherent cells was about 1 × 10^7^. As described in previous experiments, typical cell surface markers have been analyzed using flow cytometry to determine ERCs. It is worth noting that in order to ensure the uniformity of the cell function and the reliability of the results, the ERCs used in the treatment of mouse sepsis were all 4^th^ passage cells from the same volunteer.

### 2.3. Experimental Groups and Model Establishment

Mice were randomly divided into 5 groups (*n* = 6/group), including group A, normal control; group B, mice with no treatment (untreated); group C, mice treated with unmodified ERCs; group D, mice treated with AMD3100-pretreated ERCs; and group E, mice treated with SDF-1-pretreated ERCs. Through preexperiment and confirmation, the sepsis model was established by the intraperitoneal injection of PBS diluted lipopolysaccharide (LPS) (10 mg/kg) (Solarbio, Beijing, China) [24], and the normal control group received an equal amount of PBS. Unmodified ERCs were collected from the culture medium and mixed with PBS before injection. For AMD3100-pretreated ERCs, ERCs were cocultured with AMD3100 (an antagonist of CXCR4 receptor, 5 mg/kg) (Selleckchem, Shanghai, China) solution for 30 minutes before injection, as described by previous literature [9] and product specifications. For SDF-1-pretreated ERCs, ERCs were cocultured with SDF-1 solution (50 ng/ml) (PeproTech, Rocky Hill, USA) for 72 hours before injection, which can maximize the CXCR4 expression stimulated by SDF-1 coculture on the surface of ERCs, as verified previously [9]. In addition, the ERCs after coculture with SDF-1 and AMD3100 had been washed with PBS five times to eliminate the effects of residual SDF-1 and AMD3100 on the experiment. ERCs were used at 1 × 10^6^ cells/mouse by tail vein injection at 1 hour after model establishment. The mice were sacrificed at 24 hours after sepsis model establishment to collect blood samples and organs. All procedures were as gentle as possible to minimize pain and injury in mice. Blood samples were centrifuged at 3000 rpm for 10 minutes at 4°C to collect serum. Serum and organ samples were stored at -80°C until use for this study. Specimens used to make pathological sections were stored in formalin solution.

### 2.4. Determination of CXCR4 Expression on ERCs

To investigate the effect of SDF-1 on the expression of CXCR4 on ERC surface, ERCs, which had been divided into 24-well plate (5 × 10^4^ cells/well), were cocultured with two concentrations of SDF-1 (0 ng/ml, 50 ng/ml) for 72 hours at 37°C (*n* = 6/concentration). And as verified previously, SDF-1 at 50 ng/ml can maximize the CXCR4 expression stimulated by SDF-1 coculture on the surface of ERCs [9]. Then, ERCs were labeled with CXCR4 antibody (anti-CD184-PE, BioLegend, San Diego, USA), and the percentage of CXCR4^+^ ERCs was measured using flow cytometry.

### 2.5. Evaluation of Symptoms

The murine sepsis score (MSS) evaluation system as mentioned previously [25] includes seven aspects: appearance, level of consciousness, activity, response to stimulus, eye condition, respiratory rate, and respiratory quality. Each aspect is evaluated from 0 to 4 points, and the final score is the sum of the seven aspects (range, 0-28). The MSS scoring was performed by two experimenters every 4 hours, and the rectal temperature of the mice was recorded simultaneously. The survival condition of mice was recorded every two hours. The temperature of the mice was measured by using a mouse-specific rectal temperature measurement instrument, and the measurement operation was as gentle as possible to minimize the pain and damage to the mice.

### 2.6. Enzyme-Linked Immunosorbent Assay (ELISA)

Serum and homogenate of liver and lung tissues were used to measure protein levels of TNF-*α*, IL-1*β*, IL-4, and IL-10 by the ELISA kit (DAKEWE, Shenzhen, China). According to the instruction of the ELISA kit, each sample was added to three wells to minimize experiment error. The detection antibody, horseradish peroxidase, color developer, and reaction terminator were added in order. The absorbance at 450 nm was finally read, with a reference wavelength of 620 nm. The concentration of each sample was calculated based on the standard curve.

### 2.7. Quantitative Real-Time PCR (qRT-PCR)

Total RNA was extracted from liver and lung tissues using an animal tissue RNA extraction kit (TIANGEN BIOTECH, Beijing, China) according to the manufacturer's instruction. The extracted RNA was reverse transcribed into cDNA by using the FastKing one-step kit (TIANGEN BIOTECH, Beijing, China). The purity and quality of RNA and cDNA were evaluated by the absorbance at 260 nm and 280 nm using an ultraviolet spectrophotometer. qRT-PCR was performed using the RealUniversal Color PreMix (SYBR Green) kit (TIANGEN BIOTECH, Beijing, China) to assess the expression of target genes. The relative expression of target genes was analyzed by the 2^-*ΔΔ*CT^ method. The sequences of the gene-specific primers are shown in Table 1.

### 2.8. Measurement of T-SOD Activity and MDA Concentration

Parts of the liver tissues of each group cryopreserved at -80°C were made into tissue homogenate, and tissue lysate was added, followed by centrifugation at 3000 rpm for 10 minutes at 4°C to acquire supernatants which were tested for total superoxide dismutase (T-SOD) activity and malondialdehyde (MDA) concentration. The absorbance at 450 nm was measured, and the quantification criteria and procedures were performed according to the manufacturer's instructions.

### 2.9. Histology

The livers and lungs fixed in 10% formalin were embedded in paraffin. Sections were stained with hematoxylin and eosin (H&E) and evaluated by two pathologists blinded to the experimental groups. Liver damage was analyzed by semiquantitative scoring based on the following histological features: necrosis, sinus congestion and edema, lipid vacuoles, and infiltration of hyperemia and inflammatory cells. The scoring criteria include the following: 0 point = normal, 1 point = minimal changes (<25% involvement), 2 points = mild changes (25–50% involvement), 3 points = significant changes (50–75% involvement), and 4 points = severe changes (>75% involvement). The four variables were added to represent the total liver injury score (range, 0-16) [26]. The lung scoring system includes four criteria: edema, alveolar and interstitial inflammation, alveolar and interstitial hemorrhage, and necrosis. And the evaluation was also based on the 0 to 4 scoring criteria [27].

### 2.10. Immunohistochemistry

The specimen sections were incubated overnight at 4°C with proliferating cell nuclear antigen (PCNA) antibody diluted at a ratio of 1 : 4000 in PBS. And the PV9000 kit (Solarbio, Beijing, China) was used to display the signal of the PCNA antibody. Finally, the sections were counterstained with hematoxylin and observed by a microscope.

### 2.11. Statistical Analysis

Statistical analysis was performed on SPSS 25 and the experiment data were expressed as mean ± standard deviation (SD). The data obtained were compared using one-way analysis of variance (ANOVA), and Mann-Whitney *U* was used for data that did not satisfy the normal distribution. It was considered statistically significant when *p* < 0.05.

## 3. Results

### 3.1. Pretreatment with SDF-1 Increased CXCR4 Expression on ERCs

**Figure 1:**
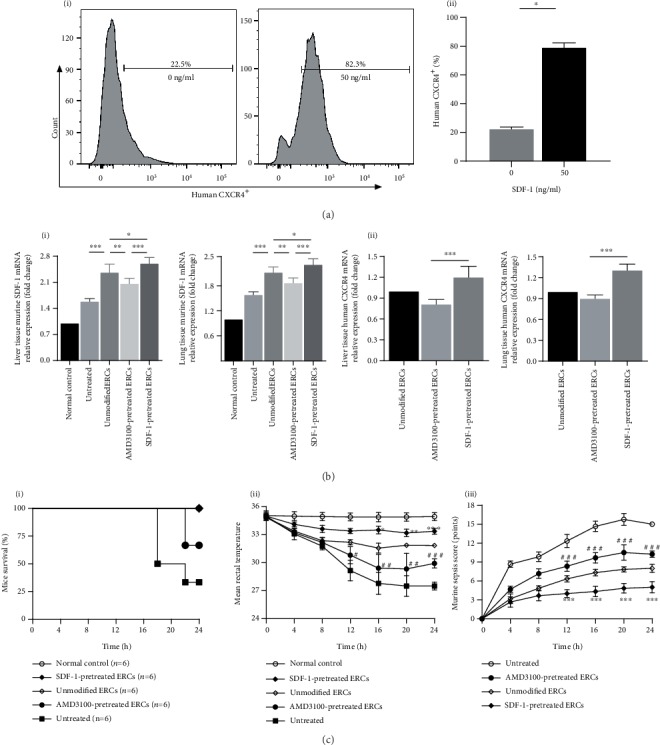
Pretreatment with SDF-1 on ERCs promoted SDF-1 and CXCR4 expression and further improved sepsis symptoms. (a) The expression of human CXCR4 on the surface of ERCs pretreated with SDF-1 at 0 ng/ml and 50 ng/ml for 72 hours. Statistical analysis was performed by Mann-Whitney *U*, *n* = 6, and ^∗^*p* < 0.05. (b) The mRNA expression levels of murine SDF-1 and human CXCR4 in liver (i) and lung (ii) tissues. Statistical analysis was performed by one-way analysis of variance (ANOVA), and the posttest was the least significant difference (LSD) test, *n* = 6. ^∗^*p* < 0.05, ^∗∗^*p* < 0.01, and ^∗∗∗^*p* < 0.001. (c) The survival rate (i), mean rectal temperature (ii), and mean murine sepsis score (iii). There was no death found in normal control group, unmodified ERC group, and SDF-1-pretreated ERC group until the end point of the observation (i). Statistical analysis was performed by one-way analysis of variance (ANOVA), and the posttest was the least significant difference (LSD) test, *n* = 6. ^∗^*p* < 0.05, ^∗∗^*p* < 0.01, and ^∗∗∗^*p* < 0.001 (SDF-1-pretreated ERC group vs. unmodified ERC group). ^#^*p* < 0.05, ^##^*p* < 0.01, and ^###^*p* < 0.001 (AMD3100-pretreated ERC group *vs.* unmodified ERC group). Bar graphs represent mean ± SD. Abbreviation: ERCs: endometrial regenerative cells.

To confirm the effect of SDF-1 pretreatment on the expression of CXCR4 on ERCs surface, we detected CXCR4 expression on the surface of ERCs after 72-hour cocultured with SDF-1 *in vitro* by using flow cytometric analysis. As shown in Figure 1(a), SDF-1 at 50 ng/ml significantly increased the CXCR4 expression on the surface of ERCs (Figure 1(a), i) compared with SDF-1 at 0 ng/ml (Figure 1(a), ii, *p* < 0.05).

### 3.2. Pretreatment with SDF-1 on ERCs Increased SDF-1 and CXCR4 Expressions in Local Tissues

To further understand whether SDF-1-pretreated ERCs would affect the expression levels of murine SDF-1 and human CXCR4 in local tissues, we tested mRNA expression levels of SDF-1 and CXCR4 in mouse liver and lungs. As shown in Figure 1(b), the results showed that the mRNA expression levels of murine SDF-1 and human CXCR4 in SDF-1-pretreated ERC group were significantly higher than those in the unmodified ERC group (Figure 1(b), SDF-1, *p* < 0.05). The above results indicate that the ERCs with elevated CXCR4 expression can cause an increase in the expression levels of murine SDF-1 and human CXCR4 in local tissues.

### 3.3. Pretreatment with SDF-1 on ERCs Further Improved Sepsis-Related Symptoms in Mice

To investigate whether SDF-1-pretreated ERCs had an ameliorative effect on sepsis in mice, we documented survival, rectal temperature, and murine sepsis score- (MSS-) based sepsis-related symptoms. As shown in Figure 1(c), in the survival results, it can be seen that, compared with the unmodified ERCs, the ERCs pretreated with AMD3100 (an antagonist of CXCR4 receptor) had a relatively negative impact on the survival of sepsis mice (Figure 1(c), i). In the mean rectal temperature results at different time points, the mean temperature in SDF-1-pretreated ERC group was maintained at a relatively higher level compared with that in the unmodified ERC group (Figure 1(c), ii), and the mean temperature in AMD3100-pretreated ERC group showed a sharp decline. In the murine sepsis score (MSS) results at different time points, mice in the SDF-1-pretreated ERC group had the mildest symptoms, and the lowest scores were obtained accordingly (Figure 1(c), iii). Meanwhile, the scores of the AMD3100-pretreated ERC group were second only to those of the untreated group. The above results indicate that SDF-1-pretreated ERCs significantly further alleviate the symptoms of sepsis mice.

### 3.4. Pretreatment with SDF-1 on ERCs Further Ameliorated Pathological Changes in the Process of Sepsis

**Figure 2:**
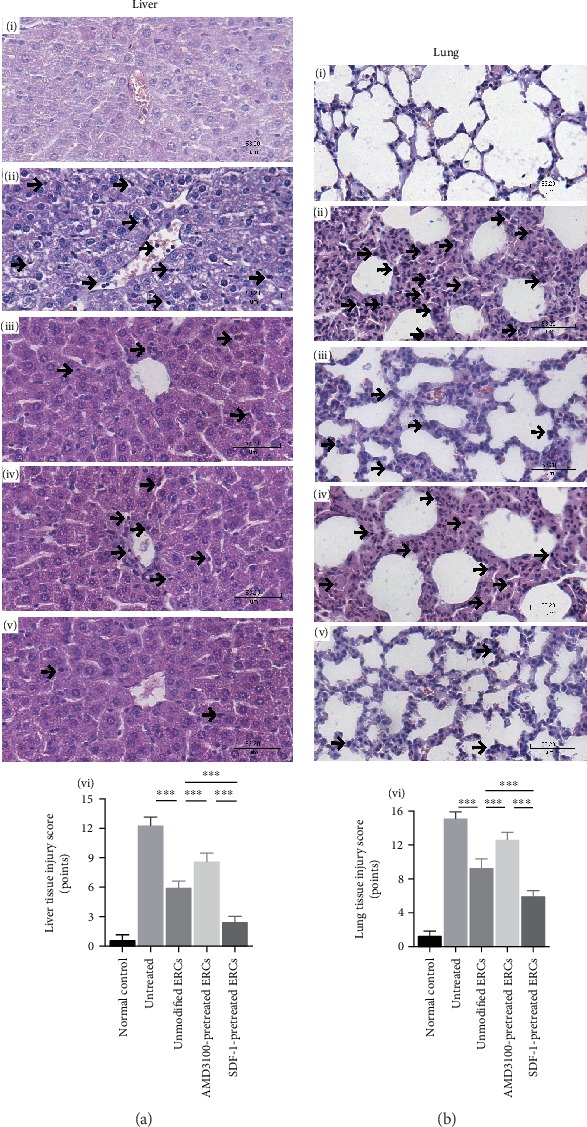
Pretreatment with SDF-1 on ERCs further ameliorated pathological changes. (a) Liver H&E-stained images are displayed at 400x magnification. Arrows indicate inflammatory cell infiltration. (i) Normal control group, (ii) untreated group, (iii) unmodified ERC group, (iv) AMD3100-pretreated ERC group, (v) SDF-1-pretreated ERC group, and (vi) the liver pathological scores. SDF-1-pretreated ERC group exhibited less ballooned hepatocytes and infiltration of inflammatory cells. (b) Lung H&E-stained images are displayed at 400x magnification. Arrows indicate inflammatory cell infiltration. (i) Normal control group, (ii) untreated group, (iii) unmodified ERC group, (iv) AMD3100-pretreated ERC group, (v) SDF-1-pretreated ERC group, and (vi) the lung pathological score. SDF-1-pretreated ERC group exhibited less edema, interstitial inflammation, and necrosis. Statistical analysis was performed by one-way analysis of variance (ANOVA), and the posttest was the least significant difference (LSD) test, *n* = 6. ^∗^*p* < 0.05, ^∗∗^*p* < 0.01, and ^∗∗∗^*p* < 0.001. Bar graphs represent mean ± SD. Abbreviation: ERCs: endometrial regenerative cells.

At the pathological level, the therapeutic effects of SDF-1-pretreated ERCs were investigated. As shown in Figure 2, the results showed that SDF-1-pretreated ERCs had better therapeutic effects than unmodified ERCs on liver and lung damages. The SDF-1-pretreated ERC group showed less necrosis, ballooned hepatocytes, and inflammatory cells infiltration in liver tissues compared with the unmodified ERC group (Figure 2(a)). In lung tissues, SDF-1-pretreated ERC group also showed less edema, interstitial dilatation, and inflammatory cells infiltration (Figure 2(b)). In contrast, the pretreatment with AMD3100 weakened the therapeutic effects of ERCs (Figures 2(a) and 2(b)). The improvement of the therapeutic effects of SDF-1-pretreated ERCs was also reflected on the pathological injury score (SDF-1-pretreated ERC group *vs.* unmodified ERC group: Figure 2(a), vi, liver injury score, *p* < 0.001; Figure 2(b), vi, lung injury score, *p* < 0.001).

### 3.5. Pretreatment with SDF-1 on ERCs Further Ameliorated Damages at a Molecular Level in the Process of Sepsis

**Figure 3:**
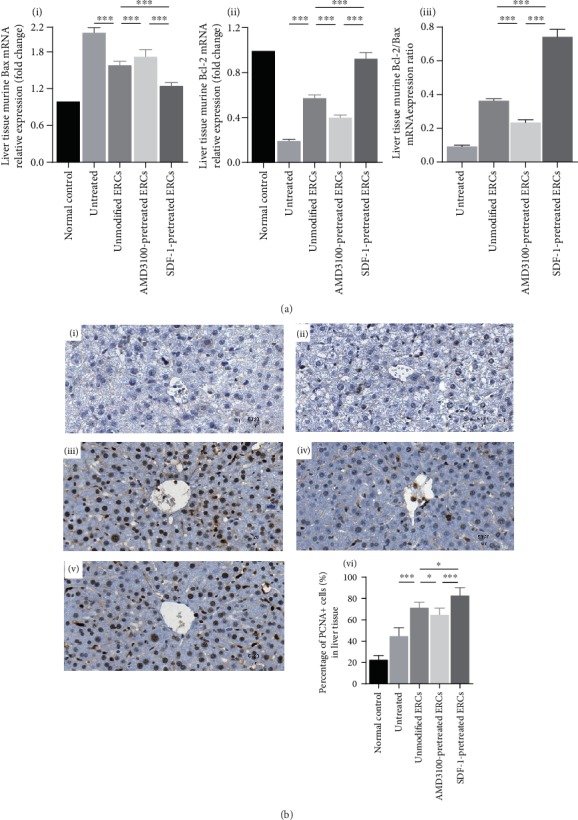
Pretreatment with SDF-1 on ERCs further ameliorated damages at molecular level in the process of sepsis. (a) The mRNA expression levels of murine Bax and Bcl-2 and Bcl-2/Bax mRNA expression ratio in liver tissue. (b) Immunohistochemical staining image of PCNA in the liver, (i) the normal control group, (ii) the untreated group, (iii) the unmodified ERC group, (iv) the AMD3100-pretreated ERC group, (v) the SDF-1-pretreated ERC group, and (vi) the percentage of PCNA-positive cells per high resolution field (400x) in the liver. Statistical analysis was performed by one-way ANOVA followed by the LSD test, *n* = 6. ^∗^*p* < 0.05, ^∗∗^*p* < 0.01, and ^∗∗∗^*p* < 0.001. Bar graphs represent mean ± SD. Abbreviation: ERCs: endometrial regenerative cells.

To determine whether SDF-1-pretreated ERCs could further reduce the damages caused by sepsis at the molecular level, we examined the mRNA levels of apoptosis-related genes Bcl-2 and Bax in liver to evaluate apoptosis. As shown in Figure 3(a), the mRNA level of the proapoptotic factor Bax increased in each group. However, compared with the unmodified ERC group, the Bax expression level in the SDF-1-pretreated ERC group was significantly lower (Figure 3(a), i, Bax, *p* < 0.001). And the Bax mRNA expression in AMD3100-pretreated ERC group was higher than that in the unmodified ERC group (Figure 3(a), i, Bax, *p* < 0.01). In the SDF-1-pretreated ERC group, the mRNA level of Bcl-2, an antiapoptotic factor, and Bcl-2/Bax mRNA expression ratio were the highest (SDF-1-pretreated ERC group *vs.* unmodified ERC group: Figure 3(a), ii, Bcl-2, *p* < 0.001; Figure 3(a), iii, Bcl-2/Bax, *p* < 0.001). In contrast, the Bcl-2 level and the Bcl-2/Bax ratio in the AMD3100-pretreated ERC group were significantly lower (AMD3100-pretreated ERC group *vs.* unmodified ERC group: Figure 3(a), ii, Bcl-2, *p* < 0.001; Figure 3(a), iii, Bcl-2/Bax, *p* < 0.001). These results indicate that SDF-1-pretreated ERCs may play a role in alleviating sepsis through reducing apoptosis in the injury organs.

To investigate the effect of SDF-1-pretreated ERCs on liver tissue cells proliferation, we performed immunohistochemistry to measure PCNA protein level in liver tissues. As shown in Figure 3(b), the higher proportion of PCNA-positive cells, the more cell proliferation. In the results, it can be seen that the proliferation activity of hepatocytes increased after ERC treatment (Figure 3(b), vi, unmodified ERC group *vs.* untreated group: PCNA^+^ cells%, *p* < 0.001), and after the treatment of SDF-1-pretreated ERCs, the cell proliferation activity was further improved (Figure 3(b), vi, SDF-1-pretreated ERC group *vs.* unmodified ERC group: PCNA^+^ cells%, *p* < 0.05). The above results indicate that SDF-1-pretreated ERCs can further ameliorate damages by promoting repair and regeneration in liver tissues.

### 3.6. Pretreatment with SDF-1 Further Enhanced the Anti-Inflammatory Effect of ERCs in the Process of Sepsis

**Figure 4:**
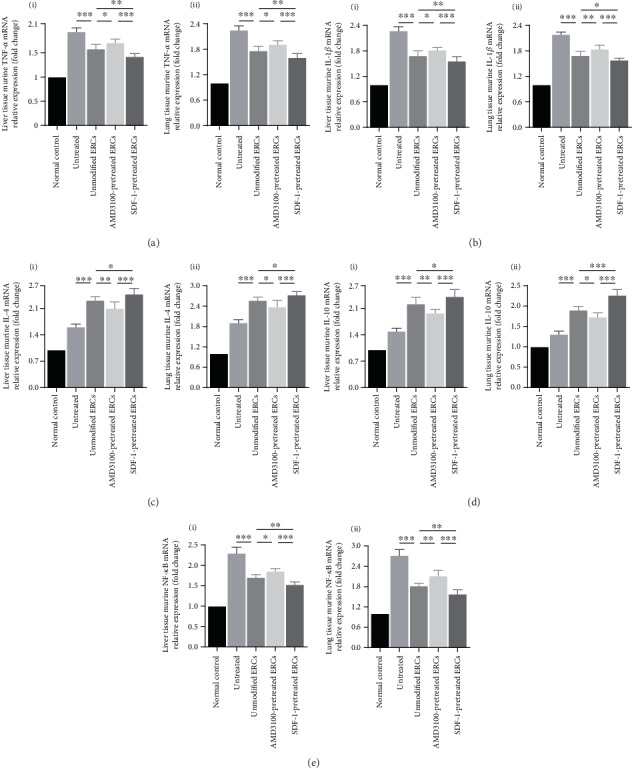
Pretreatment with SDF-1 further enhanced the anti-inflammatory effect of ERCs in the process of sepsis. The mRNA expression levels of murine TNF-*α* (a), IL-1*β* (b), IL-4 (c), IL-10 (d), and NF-*κ*B (e) in liver and lung tissues. Statistical analysis was performed by one-way ANOVA followed by the LSD test, *n* = 6. ^∗^*p* < 0.05, ^∗∗^*p* < 0.01, and ^∗∗∗^*p* < 0.001. Bar graphs represent mean ± SD. Abbreviation: ERCs: endometrial regenerative cells.

**Figure 5:**
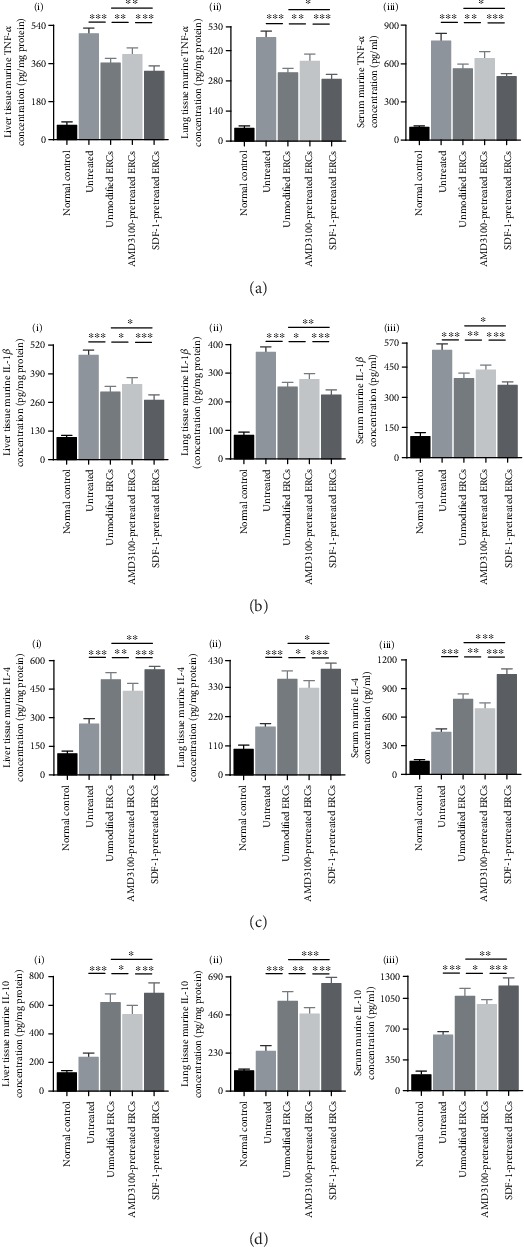
Pretreatment with SDF-1 further enhanced the anti-inflammatory effect of ERCs in the process of sepsis. The protein concentration of murine TNF-*α* (a), IL-1*β* (b), IL-4 (c), and IL-10 (d) in the liver, lung, and serum. Statistical analysis was performed by one-way ANOVA followed by the LSD test, *n* = 6. ^∗^*p* < 0.05, ^∗∗^*p* < 0.01, and ^∗∗∗^*p* < 0.001. Bar graphs represent mean ± SD. Abbreviation: ERCs: endometrial regenerative cells.

To investigate the effects of SDF-1 on the immunoregulatory ability of ERCs, we examined inflammatory factor levels in the liver, lung, and serum. As shown in Figures 4 and 5 , TNF-*α* and IL-1*β* levels were significantly lower (Figures 4(a) and 4(b), TNF-*α*, *p* < 0.001, IL-1*β*, *p* < 0.001; Figures 5(a) and 5(b), TNF-*α*, *p* < 0.001, IL-1*β*, *p* < 0.001), while IL-4 and IL-10 levels were significantly higher (Figures 4(c) and 4(d), IL-4, *p* < 0.001, IL-10, *p* < 0.001; Figures 5(c) and 5(d), IL-4, *p* < 0.001, IL-10, *p* < 0.001) in the unmodified ERC group than those in the untreated group. In the SDF-1-pretreated ERC group, TNF-*α* and IL-1*β* levels further reduced (Figures 4(a) and 4(b), TNF-*α* in the liver, *p* < 0.01, TNF-*α* in the lung, *p* < 0.05, IL-1*β*, *p* < 0.05; Figures 5(a) and 5(b), TNF-*α* in the liver, *p* < 0.01, TNF-*α* in the lung and serum, *p* < 0.05, IL-1*β* in the liver and serum, *p* < 0.05, and IL-1*β* in the lung, *p* < 0.01), while IL-4 and IL-10 levels were further increased (Figures 4(c) and 4(d), IL-4, *p* < 0.05, IL-10 in the liver, *p* < 0.05, IL-10 in the lung, *p* < 0.001; Figures 5(c) and 5(d), IL-4 in the liver, *p* < 0.01, IL-4 in the lung, *p* < 0.05, IL-4 in the serum, *p* < 0.001, IL-10 in the liver, *p* < 0.05, IL-10 in the lung, *p* < 0.001, and IL-10 in the serum, *p* < 0.01), as compared with the unmodified ERC group. Above potent immunoregulatory effect was significantly attenuated in the AMD3100-pretreated ERC group (Figures 4 and 5, AMD3100-pretreated ERC group *vs.* unmodified ERC group). As shown in Figure 4(e), in SDF-1-pretreated ERC group, there was a significant decrease of NF-*κ*B mRNA expression in the liver and lung tissues (Figure 4(e), SDF-1-pretreated ERC group *vs.* unmodified ERC group: NF-*κ*B, *p* < 0.01). The results indicate that the SDF-1 pretreatment significantly enhance the anti-inflammatory and immunoregulatory properties of ERCs in the process of sepsis.

### 3.7. Pretreatment with SDF-1 Further Enhanced the Antioxidative Effect of ERCs in the Process of Sepsis

**Figure 6:**
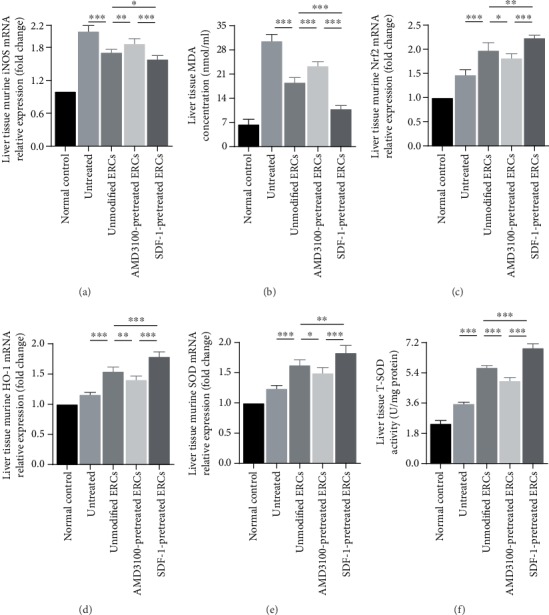
Pretreatment with SDF-1 further enhanced the antioxidative effect of ERCs in the process of sepsis. The mRNA expression levels of murine iNOS (a), Nrf2 (c), HO-1 (d), SOD (e), T-SOD activity (f), and MDA concentration (b) in liver. Statistical analysis was performed by one-way ANOVA followed by the LSD test, *n* = 6. ^∗^*p* < 0.05, ^∗∗^*p* < 0.01, and ^∗∗∗^*p* < 0.001. Bar graphs represent mean ± SD. Abbreviation: ERCs: endometrial regenerative cells.

To determine the role of the SDF-1 pretreatment on ERCs in oxidative stress, we examined the mRNA expression of inducible nitric oxide synthase (iNOS), heme oxygenase 1 (HO-1), superoxide dismutase (SOD), nuclear factor erythroid 2-related factor 2 (Nrf2), SOD activity, and MDA concentration in liver tissue. As shown in Figure 6, in the untreated group, iNOS mRNA level and MDA (oxidative stress product) concentration were both increased (Figures 6(a) and 6(b), untreated group *vs.* unmodified ERC group: iNOS, *p* < 0.001; MDA, *p* < 0.001). ERCs showed a significantly inhibitory effect on the elevation of iNOS mRNA level and MDA concentration, and the SDF-1 pretreatment enhanced the ability of ERCs to downregulate iNOS mRNA level and MDA concentration (Figures 6(a) and 6(b), SDF-1-pretreated ERC group *vs.* unmodified ERC group: iNOS, *p* < 0.05; MDA, *p* < 0.001). Compared with the unmodified ERC group, the mRNA levels of the antioxidative stress factors Nrf2, HO-1, SOD, and total SOD activity were significantly increased in the SDF-1-pretreated ERC group (Figures 6(c)–6(f), Nrf2, *p* < 0.01; HO-1, *p* < 0.001; SOD, *p* < 0.01; T-SOD, *p* < 0.001). Moreover, antioxidative stress factors expression and total SOD activity in AMD3100-pretreated ERC group were significantly lower than those in unmodified ERC group (Figures 6(c)–6(f), AMD3100-pretreated ERC group *vs.* unmodified ERC group: Nrf2, *p* < 0.05; HO-1, *p* < 0.01; SOD, *p* < 0.05; T-SOD, *p* < 0.001). These data indicate that the SDF-1-pretreated ERCs can significantly regulate oxidative stress disorder in liver tissue.

## 4. Discussion

SDF-1/CXCR4 pathway plays a vital role in mesenchymal stem cells migration, secretion [28], and immune regulation [29]. It has been also proven in several studies that human ERCs are able to migrate to the injured sites in mice and play a therapeutic role in immunoregulation [9, 13]. However, the role of SDF-1/CXCR4 pathway in the therapeutic effects of ERCs needs to be elucidated in more disease models. In the current study, we have demonstrated that, in a mouse sepsis model, the immunoregulatory and antioxidative abilities of the ERCs were significantly enhanced by SDF-1 pretreatment. Compared with the unmodified ERCs, the beneficial therapeutic abilities of the ERCs pretreated with AMD3100 (a potent antagonist of CXCR4 receptor, can effectively block the function of SDF-1/CXCR4 signaling pathway) were significantly weakened.

In the current study, the *in vitro* experiments showed that SDF-1 pretreatment could increase the expression of CXCR4 on the surface of ERCs (Figure 1(a)), and the *in vivo* experiments confirmed that the high expression of CXCR4 on human ERCs could persist in mice (Figure 1(b)). Under the chemotaxis of SDF-1 produced at the injured sites, CXCR4-expressing cells can migrate to the corresponding sites [28]. The increased CXCR4 expression on ERCs surface promotes the chemotaxis and migration of ERCs to inflammatory injury sites [9], which ensures the therapeutic effects of ERCs to be more fully achieved at the same therapeutic dose. What is more, it has been proven that ERCs can also secrete high levels of SDF-1, which is 9824 ± 1700 pg/ml in culture supernatant measured by ELISA [11]. In our experiments, the results showed that the expression level of SDF-1 in injury organs was significantly increased in SDF-1-pretreated ERC group (Figure 1(b)). Studies have shown that SDF-1 can promote the conversion of macrophages to the anti-inflammatory type 2 cell phenotype [30] and can limit the development of inflammatory responses by constraining monocytes at injury sites [31]. In the current study, we found that ERCs with overexpressed CXCR4 could reach the injury sites under chemotaxis by SDF-1 and could secrete SDF-1 to recruit more ERCs to regulate immune imbalance and relieve tissue damage.

In a myocardial ischemia-reperfusion model, the excessive activation of SDF-1/CXCR4 pathway maintains the mitochondrial membrane potential and membrane permeability of cardiomyocytes to resist oxidative stress damage [32]. Moreover, there has been a study showing that the activation of the SDF-1/CXCR7 pathway (another signaling pathway mediated by SDF-1, but functionally different from SDF-1/CXCR4) of EPCs generates a significant resistance to destructive damage caused by oxidative stress [33]. Furthermore, the blocking of the SDF-1/CXCR7 axis or the knockout of Nrf2 deprives EPCs of resistance to oxidative stress injury [33]. In the current study, in the SDF-1-pretreated ERC group, Nrf2, HO-1, SOD mRNA expression, and T-SOD activity showed a significant increase (Figure 6), meanwhile, iNOS mRNA expression and MDA concentration showed a significant decrease (Figure 6). It is worth noting that iNOS plays an important role in promoting oxidative stress damage [34], and MDA is an oxidative stress product [35]. Taken together, SDF-1 pretreatment significantly enhanced the regulation ability of ERCs in oxidative response disorder. SDF-1 secreted by ERCs may interact with the SDF-1/CXCR4 axis and SDF-1/CXCR7 axis in local tissues to exert antioxidative stress effect.

SDF-1 mainly achieves tissue repair by promoting angiogenesis in injury areas through recruiting bone marrow-derived cells with angiogenic activity in the peripheral blood [36]. Moreover, elevated CXCR4 expression on ERCs surface promotes the migration of ERCs to injury sites [9], which enables ERCs to play a role in tissue repair. Previous research has shown that the PCNA protein level is positively correlated with the ability of tissue repair [37]. In the SDF-1-pretreated ERC group, the immunohistochemistry results showed that the percentage of PCNA-positive cells was significantly higher than that of other groups (Figure 3), indicating that SDF-1 enhances the ability of ERCs to promote cell proliferation and tissue repair. Moreover, PCNA also plays a role in the regulation of apoptosis. The binding of PCNA to procaspase-3/8/9 can inhibit their activation and prevent apoptosis [38]. In addition, SDF-1 can protect cells from apoptosis through decreasing Bax protein and increasing Bcl-2 protein and Bcl-2/Bax ratio [39]. Furthermore, increased expression of Bcl-2 and decreased activity of caspase-3 can significantly improve survival in septic mice [40]. In summary, the secretion of SDF-1 by ERCs may be one of the mechanisms for ERCs to relieve tissue injury.

In the current study, we mainly discussed the positive therapeutic effect of SDF-1-pretreated ERCs on the liver and lung in an infectious disease; however, we know that sepsis is a systemic inflammatory disease that involves many organs of the respiratory, circulatory, digestive, and nervous systems. We believe that ERCs could also have a positive effect on the relief of damage to organs other than the liver and lung. Considering the significant improvement of SDF-1 on the therapeutic effects of human ERCs, SDF-1-pretreated ERCs could also play a significant role in treating other diseases such as autoimmune hepatitis, acute pancreatitis, and acute myocardial infarction. Moreover, anti-infective therapy is an important strategy in the treatment of clinical sepsis, and the use of antibiotics can directly clear the pathogen. The role of SDF-1-pretreated ERCs in the treatment of sepsis is mainly to regulate the disordered inflammatory and oxidative stress responses. Therefore, we speculate that the combined effects of SDF-1-pretreated ERCs and antibiotics in the clinical application are coordinated rather than conflicting. However, the feasibility and safety of combined use of SDF-1-pretreated ERCs and antibiotics in sepsis need further investigation.

In terms of safety for clinical applications, ERCs have been proven safe in the treatment of human multiple sclerosis [41]. Furthermore, during the follow-up of more than one year, no adverse immune reaction and treatment-related adverse reactions occurred in patients treated with intravenous or intrathecal ERCs injection [41]. In a case report of allogeneic ERCs combined with cord blood CD34 positive cells for the treatment of ischemia cardiomyopathy, there is also no treatment-related adverse reactions reported [42]. From the existing knowledge about ERCs, we can understand that the safety of ERCs application in human body can be basically guaranteed. However, ERCs are still a cell type that has not been clinically validated on a large scale, and its safety needs to be further clarified.

The current study demonstrates that SDF-1 has markedly improved the therapeutic effects of ERCs in a mouse sepsis model in alleviating sepsis-related symptoms, reducing tissue damage, regulating inflammatory imbalance, and relieving oxidative stress. However, it still requires further research to elucidate more detailed mechanisms for the improved therapeutic effects of SDF-1-pretreated ERCs. With the further research on ERCs and the further development of cell therapy, SDF-1- pretreated ERCs will provide a new perspective for the clinical treatment of sepsis or more diseases.

## 5. Conclusions

In this study, we have demonstrated that SDF-1 pretreatment has a significant improvement in the therapeutic effects of ERCs in a mouse sepsis model, which reflects mainly on alleviating sepsis-related symptoms, reducing tissue damage, regulating inflammatory imbalance, and relieving oxidative stress. The increase of CXCR4 expression on the surface of ERCs induced by SDF-1 pretreatment is the key to the improvement in therapeutic effects of ERCs. However, more detailed mechanisms for the improvement in therapeutic effects of SDF-1-pretreated ERCs require further research to be elucidated. Furthermore, SDF-1-pretreated ERCs are expected to be in wider clinical application in the future.

## Figures and Tables

**Table 1:** Gene-specific primers for qRT-PCR.

Gene	Primers (5′-3′)
GAPDH	Forward: AGGTCGGTGTGAACGGATTTG
	Reverse: TGTAGACCATGTAGTTGAGGTCA
HO-1	Forward: AAGCCGAGAATGCTGAGTTCA
	Reverse: GCCGTGTAGATATGGTACAAGGA
SOD	Forward: CAGACCTGCCTTACGACTATGG
	Reverse: CTCGGTGGCGTTGAGATTGTT
iNOS	Forward: GTTCTCAGCCCAACAATACAAGA
	Reverse: GTGGACGGGTCGATGTCAC
Nrf2	Forward: TCTTGGAGTAAGTCGAGAAGTGT
	Reverse: GTTGAAACTGAGCGAAAAAGGC
NF-*κ*B	Forward: ATGGCAGACGATGATCCCTAC
	Reverse: TGTTGACAGTGGTATTTCTGGTG
TNF-*α*	Forward: CCCTCACACTCAGATCATCTTCT
	Reverse: GCTACGACGTGGGCTACAG
IL-1*β*	Forward: TTCAGGCAGGCAGTATCACTC
	Reverse: GAAGGTCCACGGGAAAGACAC
IL-4	Forward: GGTCTCAACCCCCAGCTAGT
	Reverse: GCCGATGATCTCTCTCAAGTGAT
IL-10	Forward: GCTCTTACTGACTGGCATGAG
	Reverse: CGCAGCTCTAGGAGCATGTG
Bcl-2	Forward: GTCGCTACCGTCGTGACTTC
	Reverse: CAGACATGCACCTACCCAGC
Bax	Forward: TGAAGACAGGGGCCTTTTTG
	Reverse: AATTCGCCGGAGACACTCG
SDF-1	Forward: TGCATCAGTGACGGTAAACCA
	Reverse: TTCTTCAGCCGTGCAACAATC
CXCR4	Forward: ACGCCACCAACAGTCAGAG
	Reverse: AGTCGGGAATAGTCAGCAGGA

## Data Availability

The data used to support the findings of this study are available from the corresponding author upon request.
